# Tendency to Insanity at Childbirth

**Published:** 1844-03-23

**Authors:** J. R. Cormach


### TENDENCY TO INSANITY AT CHILDBIRTH.

Parturition and delivery are not uncommon causes of temporary insanity. In accusation of infanticide against the mother temporary insanity may, therefore, be pleaded in defence, and in some instances be a perfectly valid ground of exculpation, the more especially as infanticidal mania is one of the most common forms in which temporary insanity manifests itself in connection with delivery. Rabbits, cats, bitches, swine, and some birds are peculiarly subject to have the maternal instinct to protect, converted into a furious passion to destroy their offspring. (See

Pierquin, De la Folie des Animaux," tom. ii.) Even in natural labour, especially in first births, the mental faculties are frequently affected. Moral or physical causes, either singly or combined, convert this unsettled state of mind into actual mania; and both causes are peculiarly apt to be in operation in unmarried mothers against whom charges of infanticide are most common.—*Dr. J. R. Cormack.*

